# Rare case of an adrenocortical neoplasm: A case report and review of literature

**DOI:** 10.3892/ol.2014.2584

**Published:** 2014-10-02

**Authors:** ROSSELLA ANGOTTI, FRANCESCO MOLINARO, ANNA LAVINIA BULOTTA, GIOVANNI DI MAGGIO, ELISA BRANDIGI, MARIO MESSINA

**Affiliations:** Department of Pediatric Surgery, University of Siena, Siena 53100, Italy

**Keywords:** adrenal glands, neoplasm, infant

## Abstract

Adrenocortical neoplasms (ACNs) are rare and poorly characterized in infants. The true incidence of ACNs is not well known and it appears to vary substantially across different geographical areas. ACNs are more common in females and two peaks of incidence have been identified: The first year of life and between the age of nine and 16 years. Due to the heterogeneity and rarity of ACNs, their pathological and prognostic classification is challenging. The current study describes the case of a seven-year-old male, who presented to the Department of Pediatric Surgery, University of Siena (Siena, Italy) with a feminization syndrome and increased somatic growth that was associated with a unilateral adrenal mass, which was diagnosed by magnetic resonance imaging. Surgical excision of the mass was performed and histological analysis determined that it was an ACN, with a low risk of malignity; however, the pathological classification of the tumor was challenging. At present, the future behavior of ACNs is unpredictable. Therefore, increasing the knowledge surrounding this type of tumor may aid in its diagnosis, treatment and prognosis. Due to the rarity of pediatric ACNs, no single pediatric oncology center has acquired extensive experience treating this type of tumor. Thus, the initiation of an international tumor registry may aid with the management of patients presenting with ACNs.

## Introduction

Adrenocortical neoplasms (ACNs) arise from the adrenal cortex and are classically considered to be epithelial tumors (carcinoma or adenoma) (1). This class of tumors is rare and poorly characterized in infants. The tumors exhibit rare clinical and biological features, which differ from those observed in other pediatric carcinomas or the same neoplasms in adults. ACNs are more common in females and two peaks of incidence have been identified, in the first year of life and between the age of 9 and 16 years. The manifestations of ACNs are dependent on the secretion of adrenocortical hormones by the tumor. Cushing’s syndrome if the most common manifestation, hwoever, feminizing and masculinizing syndromes are less common and Conn syndrome, with aldosterone production, is rare. ACNs usually secrete several hormones and thus, present the symtpoms of multiple syndromes. Non-functional tumors, which are neoplasms which do not produce adrenocortical hormones, account for ~10% of pediatric cases (1). The diagnosis of ACN is challenging and it is determined according to the gross and histological appearance of tissue samples obtained during surgery (2–5). Therefore, the role of the pediatric surgeon in this type of tumor is considered to be significant. The pathological classification of pediatric ACNs is difficult and experienced pathologists may find it challenging to differentiate between a carcinoma and an adenoma. The aim of this report was to describe the case of a seven-year-old male that presented with an ACN, and to analyze the management of this type of tumor, focusing on the onset, the delay in its diagnosis and the difficulties in its histological classification. Written informed consent was obtained from the patient’s family.

## Case report

**Figure 1:**
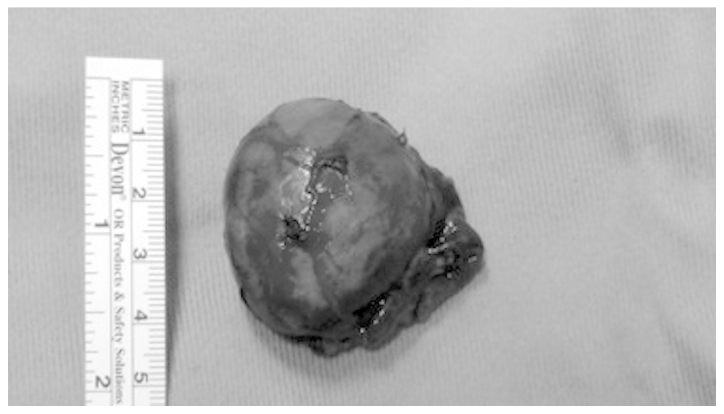
Intraoperative image of the excised tumor.

In September 2012, a seven-year-old male was admitted to the Department of Pediatric Surgery, University of Siena (Siena, Italy) with a feminization syndrome, which presented as bilateral gynecomastia. Increased somatic growth was observed (bone age was advanced by more than two years) and a unilateral adrenal mass was diagnosed by ultrasound (USS). The symptoms had begun approximately eight months prior to admission, however, the patient had received treatment for an endocrinological disease. The patient’s plasma testosterone was lower than normal (0.57 ng/ml, normal range, 2.7–10.9 ng/ml) and the androstenedione level was within the normal range (3.08 ng/ml, normal range, 0.4–3.1 ng/ml). The plasma estradiol level (54.6 pg/ml, normal range, <50 pg/ml) was greater than normal. The presence of the mass was confirmed by magnetic resonance imaging (MRI), which revealed a unilateral lesion of the adrenal gland, measuring ~3.8×2.7×3.2 mm. The lesion was well-defined and no additional abdominal abnormalities were present. Furthermore, no lymphadenopathies were observed. The results for metanephrines and catecholamines obtained from a 24-h urine test were negative. In addition, a computed tomography scan revealed that there was no involvement of the chest. Surgical excision of the mass was performed via an open anterior transperitoneal approach and the entire adrenal gland was removed (Fig. 1). A biopsy was performed to remove three lymph nodes, as recommended by TREP guidelines (4), however, they did not appear to be grossly involved. The histological analysis identified that the lymph nodes were not involved and determined that the mass was an ACN, with a low risk of malignity. There were no complications during or post-surgery, therefore, the postoperative care was normal and the patient was discharged on postoperative day six. The initial follow-up was conducted one month following surgery, and an abdominal USS and hormone profile were performed. The abdominal USS was normal and the hormone profile revealed reduced levels of plasma testosterone (<0.1 mg/ml, normal range, 2.7–10.9 ng/ml), androstenedione (<0.3 ng/ml, normal range, 0.4–3.1 ng/ml) and estradiol (<20.0 pg/ml, normal range, <50 pg/ml) levels. However, the cortisol level (23.6 pg/ml, normal range, 10–52 pg/ml) was normal. The next surgical follow-ups are expected to be two and six months following surgery.

## Discussion

The adrenocortical neoplasm (ACN) is a rare type of pediatric tumor and represents <0.5% of all childhood neoplasms and 6% of all pediatric adrenal tumors (1,3–5). The true incidence of these tumors is not well known and it appears to vary substantially across geographical areas. In the USA, ~10,000 new cases of ACN are diagnosed annually in patients aged <20 years (1). In Southern Brazil (São Paulo and Parana), however, the incidence is approximately >10 times this (1); however, the cause of this increased rate has not been identified. Furthermore, this type of tumor is more common in females, although the reason for this remains unknown (6). Two peaks of incidence of ACN have been identified: The first year of life and between the ages of nine and 16 years. Predisposing inherent genetic factors have also been identified in ~50% of infants exhibiting ACNs. Furthermore, two genetic syndromes, Li-Fraumeni and Beckwith Wiedemann (7–9), are associated with these tumors and its frequency of presentation in individuals with these syndromes is >100 times that of the general population (7). Additionally, the association between ACNs and environmental factors, such as prenatal exposure to carcinogens or fetal alcohol syndrome, has been reported in previous studies, however, was difficult to verify as these factors may also be associated with other neoplasms (11,12). The patient described in the current report was a seven-year-old Italian male, without any associated syndrome or antenatal history of exposure to carcinogenic factors. The patient was admitted to the Department of Pediatric Surgery eight months following the onset of symptoms of bilateral gynecomastia (indicating a feminization syndrome) and increased somatic growth. Initially, the patient received treatment for an endocrinological disease, and only following an USS that revealed the adrenal mass, was the patient referred to the Department of Pediatric Surgery. This is consistent with previously reported cases that describe the secretion of adrenocortical hormones by the tumor as the initial manifestation of the presentation of ACNs (13–15). Cushing’s syndrome is the most frequent symptom of raised hormone levels; however, the feminizing and masculinizing syndromes are less common and present as the initial manifestation of ACN in only 2.2% of cases (16). Furthermorem, Conn’s syndrome, which produces aldosterone, is rare. However, although the clinical manifestations of one endocrine syndrome may predominate, ACNs often secrete a variety of hormones and, thus, present the signs and symptoms of multiple syndromes (mixed forms).

Non-functional tumors comprise ~10% of pediatric cases, worldwide. However, a significant clinical manifestation that is observed in patients with functioning ACNs, and androgen and estrogen overproduction, is growth disturbance in terms of overgrowth. Generally, an infant exhibiting this condition presents with increased somatic growth for their chronological age, however, are generally healthy. This clinical effect is described by numerous studies (13–15). Hauffa *et al* (15), demonstrated increased growth in 10 infants aged 0.8–11.8 years who exhibited adrenocortical tumors and hormone overproduction. The implications of recognizing this as a symptom of ACN are significant, and may facilitate the early diagnosis of the tumor prior to the onset of other tumor-associated symptoms, and increase the possibility of avoiding long-term effects on final adult height (although it is currently unclear if these factors are correlated).

Due to the rarity of pediatric ACNs, the majority of pediatric onoclogy centers have reported observations of only a small number of patients over several years and there are few multicenter, long-duration studies with a large series. Lefevre *et al* (16) reported 42 infants treated in various French hospitals over a 22-year period. Ribeiro *et al* (17–19) reported the results of infants with ACNs treated at a single institution in Southern Brazil over a duration of ~10 years. In another study, Liou and Kay (20) summarized the results of 412 patients from a variety of published series, predominantly from the USA. Michalkiewicz *et al* (3), provided a descriptive analysis of 254 patients that were registered on the International Pediatric Adrenocortical Tumor Registry (3). The current study followed the guidelines proposed by TREP to determine the diagnostic procedures and treatment management (4). These guidelines were developed by an Italian research group that aim to establish guidelines for the diagnosis and treatment of rare types of pediatric tumor. The diagnosis in the current study was determined by abdominal USS and MRI, in addition to conducting a complete hormone profile (as recommended by the TREP guidelines). The role of the pediatric surgeon is particularly significant in the pathology of ACNs and surgical management must be considered for all cases of childhood ACN, although the optimum treatment strategy remains unknown. The literature surrounding the treatment of pediatric ACN cases is poor, while it is well clarified in adult patients (18). However, there is currently no gold standard approach. Numerous studies have proposed open transperitoneal resection as it is associated with a decreased risk of bleeding, improved visualization of the tumor boundaries and it allows for the inspection and biopsy of the contralateral adrenal and adjacent lymph nodes (1,2,4). Thus, an open transperitoneal resection was conducted in the present case. However, as described by Cobb *et al* (21) a laparoscopic approach (via a transperitoneal or retroperitoneal route) may be an alternative option for a highly experienced surgeon treating older patients (>13 years) that present with a low probability of malignancy and small tumors (<6 cm). It is evident that determination of the surgical approach is influenced by the surgeon’s experience, however, irrespective of the type of surgery, all surgeons must endeavor to achieve complete removal of the tumor, potentially at the expense of adjacent structures, and perform a regional lymph node biopsy (even when lymph node involvement is not apparent).

The histological features of this type of tumor remain controversial. The pathologist usually determines the definitive diagnosis and prognosis of the tumor; however, in pediatric adenocarcinoma, the role of the pathologist is different. Due to the rarity of pediatric ACN, the experience of patholgists is limited and thus, it is difficult for pathologists to establish a diagnosis. The histological features are not considered to be markers of malignancy in infants, and due to the heterogeneity and rarity of ACNs, the prognostic factors have been difficult to establish. Prognostic factors are based on the TREP pathway, which includes the following: The radicality of exeresis; the presence of metastasis; the weight of the mass, which is considered to be the predominant prognostic element (cut off, <150 g); the duration of symptoms (cut off, six months); age of the infant (<3 years indicates improved prognosis); and histological features (4). The survival rate of ACNs is between 15 and 70%. However, prognosis is dependent on numerous factors. A young patient (<3 years), presenting with symptoms within six months of onset, exhibiting a small mass (<150 g) who receives an open complete resection, and demonstrates negative results from histological analysis of the lymph nodes and does not exhibit metastasis is a typical case with an optimal prognosis. Notably, pediatric adrenal tumors are associated with an improved prognosis when compared with the same masses in adults. The staging of ACN in infants varies from that used for adults and it is based on the size of the mass, the evidence of invasion or regional/distant metastasis, the radical removal of the tumor and the normalization of hormone levels following surgery (5,18,23). The patient in the current study had a stage 1 ACN (4). However, the risk stratification system, which was developed to guide physicians in treatment planning, is considered to be more important than tumor staging. The risk groups are based upon the success of the administered treatment and the survival rates. Patients are assigned to one of the three groups: Low, intermediate or high risk (18). In the current study, the patient was assigned to the low risk group. The benefit of risk grouping is that it provides the patient with the optimal treatment plan, while minimizing the requirement for toxic therapies. As described in the TREP guidelines, the postoperative evaluation involves USS or MRI of the site of the excised tumor and hormone profiling (4), which were conducted for the current patient two weeks following surgery and exhibited negative results. The future follow-up, which will comprise the same analysis, will be performed at two-month intervals for the initial two years, at three-month intervals in the third year, at four-month intervals in the fourth year and annually from the fifth year onwards. However, the infant in the present case was referred to the Department of Oncology to manage the medical aspects of the pathology.

In conclusion, the current report may be beneficial in increasing the knowledge of ACNs. The present case of ACN in an infant male was managed according to the TREP guidelines; however, an international tumor registry is proposed as it may aid with the consistent management of these types of patient worldwide. Furthermore, the present report aimed to highlight the important role of pediatric surgeons, who are required within the team that manages the pathological analysis of ACNs in infants. Additionally, we described the marginal role that pathologists have had in previous cases, which explains the challenges experienced by the pathologist involved in the present case in diagnosing this type of tumor.
